# The role of multipotent cancer associated fibroblasts in hepatocarcinogenesis

**DOI:** 10.1186/s12885-015-1196-y

**Published:** 2015-03-27

**Authors:** Caecilia Hapsari Ceriapuri Sukowati, Beatrice Anfuso, Lory Saveria Crocé, Claudio Tiribelli

**Affiliations:** 1 Department of Medicine Surgery and Health Sciences, University of Trieste, 34100 Trieste, Italy; 2 Fondazione Italiana Fegato - Italian Liver Foundation, AREA Science Park Basovizza, 34149 Trieste, Italy

**Keywords:** Hepatocellular carcinoma, Cancer-associated fibroblasts, Stroma, Stem cells, Cancer stem cells, Microenvironment

## Abstract

**Background:**

The presence of tumor supporting cells in various cancer, including in hepatocellular carcinoma (HCC), has become an important target in the study of carcinogenesis. The cancer-associated fibroblast (CAF), one of the most important cellular components in the cancer stroma, might contribute to the progression of the disease due to its plasticity, a behavior of the stem cells. In this study, we investigate the significance of the CAF and its role in the HCC progression and metastasis.

**Methods:**

Primary CAF and non-tumoral fibroblast (NTF) from nine paired HCC and distant non-tumoral liver tissues were isolated and cultured. The cells were characterized by flow cytometry, RT-PCR, anchorage-independent assay and *in vitro* cells directed trans-differentiation. Co-culture study was performed in Transwell system and xenograft assay was performed in immunodeficient mice.

**Results:**

CAF and NTF were positive for CD90, CD44, αSMA, and vimentin and negative for CD34, CD45, CD117, and CD133. When stimulated, they showed the potential to differentiate into adipocytes, osteoblasts, and pancreatic cells. When co-cultured with human HCC cell lines, CAF up-regulated gene expressions of TGFB1 and FAP of HuH-7 and JHH-6 while NTF did not induced either of the genes. Xenograft assay showed that the CAF had the capacity to enter into circulation as confirmed by RT-PCR and DNA sequencing.

**Conclusion:**

Our data provides evidence of the plasticity of the CAF and the NTF as stem cells in the process of hepatocarcinogenesis and metastasis. These cells mutually interacts with HCC cells. Their trans-differentiation flexibility may induce a switch from normal to cancerous microenvironment.

**Electronic supplementary material:**

The online version of this article (doi:10.1186/s12885-015-1196-y) contains supplementary material, which is available to authorized users.

## Background

Liver cancer is the fifth most common cancer in men and the ninth in women, and the second most common cause of cancer-related death, estimated to be responsible for around 9% of all cases in 2012 [1]. Hepatocellular carcinoma (HCC) accounts for 85% to 90% of liver cancer cases [2].

Recent studies had shown the importance of the cross talk between cancer cells and their stromal microenvironment, including in the HCC. The cancer-associated fibroblast (CAF), sometimes acknowledged as cancer stromal cell, is the most important cell type in the stroma. Previously, Mazzocca *et al.* had demonstrated an interaction between CAF and HCC cells. The CAF appeared essential for tumor growth and metastasis and the HCC cells stimulated the proliferation of the CAF. HCC invasive cells produced high levels of the connective tissue growth factor (CTGF) and generated tumor with high stromal component *in vivo.* The use of transforming growth factor beta (TGFB) inhibitor was shown to inhibit tumor specific neoangiogenesis and to interrupt their cross talk, thus inhibiting tumor progression [3,4]. Interestingly, CAF-like myofibroblastic phenotype can be originated from peritumoral tissue fibroblasts (PTF) in the presence of lysophostatidic acid (LPA) secreted by HCC cells [5].

Due to its cellular heterogeneity and various physiological functions of the liver, the cellular origin of the CAF in HCC is still unclear. They can be derived from different sources such as resident fibroblast, migrated bone-marrow stem cell, or epithelial-mesenchymal transition (EMT). Previously, it had been demonstrated that the CAF in HCC have the characteristics of the multipotent resident progenitor cells through a paracrine mechanism [6].

Despite mounting evidences on the effect of the CAF in the disease progression in other cancers, its role in hepatocarcinogenesis is still undefined. In this paper, we report the potential of the stem cells-like fibroblasts present in HCC and cirrhotic liver tissue to trans-differentiate into other cell types. Data shows that this cell population plays an important role in the maintenance and the progression of liver disease.

## Methods

### Ethics

For human samples, written informed consent was obtained from patient or by a legal representative and patient anonymity has been preserved. Investigation was conducted according to the principles expressed in the Declaration of Helsinki. For animal study, the experimental procedure study was carried out in strict accordance with the recommendations in the Guide for the Care and Use of Laboratory Animals and all efforts were made to minimize suffering. The Ethical Committee Ateneo of the University of Trieste and responsible administration of the Ministry of Health of the Republic of Italy approved the protocol (Permit number: 107/2010).

### Primary cells isolation

Paired fresh HCC and distant cirrhotic liver tissues were obtained from nine HCC patients undergoing partial hepatic resection. The ratio of female:male was 5:4, mean age 73 ± 7 years; three were hepatitis C virus (HCV) positive and six were metabolic-related HCC. None of the patients received previous liver surgery, radiofrequency, and trans-arterial chemoembolization.

Tissues were finely minced with scalpel in a tissue culture dish and enzymatically dissociated in 1 mg/mL collagenase type IV (Sigma-Aldrich, St Louis, MO, USA) at 37°C for 1 hour with frequent shaking. The activity of collagenase was blocked using phosphate saline buffer (PBS) supplemented with 10% fetal bovine serum (FBS). Single cells suspension was washed and filtered through a 40 μm cell strainer (BD Biosciences, Milan, Italy). Cells were plated on a 100 mm dish in MyeloCult® medium (StemCell Technologies, Vancouver, BC, Canada) in the presence of 1 μM hydrocortisone sodium succinate and 1% antibiotics. They were maintained in a controlled CO_2_ incubator with 37°C, 95% humidity, 5% CO_2_ with medium change every three days and sub-cultured with 0.05% trypsin in PBS when they reached 80% - 90% of confluence. Morphological homogeneity of the cells were noticed along subcultures. Cells from passages 2–6 were used for all experiments. The primary cells from HCC nodules were identified as CAF while cells from distant non-tumoral tissues as NTF (non-tumoral fibroblast).

### HCC cell lines

Human HCC cell lines HuH-7 (JCRB0403) and JHH-6 (JCRB1030) were obtained from the Japan Health Science Research Resources Bank (HSRRB, Tokyo, Japan). HuH-7 cells were grown in DMEM medium (high glucose) and JHH-6 in Williams’ E medium. Both media were supplemented with 10% FBS, 1% L-glutamine, and 1% antibiotics. All cells were maintained at 37°C in a humidified 5% CO_2_ incubator and were routinely sub-cultured with 0.05% trypsin in PBS when they reached 85% - 95% confluence.

### Flow cytometry and immunofluorescence

The presence of surface marker antigens was detected using antibodies CD90/THY1 (Stem Cell Technologies, Vancouver, BC, Canada), CD133/PROM1, CD45 (Miltenyi Biotec GmbH, Bergisch Gladbach, Germany), CD44 (Abcam, Cambridge, UK), and STRO-1 (Santa Cruz Biotechnology, Inc., Santa Cruz, CA, USA). After detachment, at least two million cells per mL were incubated with specific first antibodies for 60 minutes on ice in the dark. After two washings with PBS containing 0.5% bovine serum albumin (BSA), when necessary, the cells were incubated with fluorescence-conjugated secondary antibody for 60 minutes on ice in the dark. Flow cytometric analysis was performed immediately in a FACSCalibur flow cytometer (Becton Dickinson, NJ, USA). Ten thousands events were analysed per sample.

The presence and localization of the proteins CD90, CD44, Vimentin/Vim (Abcam), and alpha smooth muscle actin/αSMA (Dako, Glostrup, Denmark) were visualized with immunofluorescence using fluorescence microscope Leica DM2000 (Leica Camera AG, Solms, Germany). The nucleus of the cells was stained with Hoechst 33342 dye (Sigma-Aldrich).

### Total RNA isolation and reverse transcription

Total RNA was extracted using TriReagent (Sigma–Aldrich) according to the manufacture’s protocol. RNA was quantified at wavelength 260 nm in a DU®730 spectrophotometer (Beckman Coulter, Fullertone, CA, USA) and RNA purity was evaluated according the MIQE guidelines [7] by measuring the ratio A260/A280 with an appropriate purity value between 1.8 and 2.0. The integrity of RNA was assessed on standard 1% agarose/formaldehyde gel. Reverse transcription of 1 μg of total RNA was done using iScript cDNA synthesis Kit (Bio-Rad Laboratories, Hercules, CA, USA).

### Real time quantitative reverse transcription polymerase chain reaction (RTqPCR)

RTqPCR was performed according to the iQ SYBR Green Supermix protocol. PCR amplification was carried out in a 15 μL reaction volume containing 25 ng of cDNA, 1x iQ SYBR Green Supermix, and 250 nM gene specific sense and anti-sense primers and reaction was run on a Bio-Rad iQ5 real-time PCR detection system (IQ5 software version 3.1; Bio-Rad Laboratories), together with reference genes RNA18S and β-actin (ACTB). Cycling parameters were determined and analyzed using the Pfaffl modification of ΔΔCt equation with taking account to the efficiency of the reaction [8,9]. The primers for PCR were designed using software Beacon Designer Version 7.9 (Premier Biosoft International, Palo Alto, CA, USA). Primer sets were built across two exons to avoid the contamination of genomic DNA. Nucleotide BLAST was performed to check the specificity of the sequences. Melting curve analysis and agarose gel electrophoresis were carried out to assess the size of the template products. The list of the primers sequences was listed in Supplemental File (Additional file 1: Table S1). Control for of primers: total RNA extract from IHH cells for CD90; HuH-7 cells for CD133 [10,11]; Jurkat cells for CD34; and HCC tissues and blood samples for CD44, CD45, CD29, CD31, CD105, CD166, CD11B, CD13, CD19, CD29, and CD79.

### Clonogenic assay

The clonogenic capacity of the CAF in a 3-dimensional anchorage-independent matrix was performed by growing the cells at low density of 5000 cells/mL in 67% Matrigel (BD Biosciences) in growth medium without serum on a 12-well cell culture plate. The clones were observed under light microscope every three days (Nikon Eclipse TS100, Nikon Instruments, Campi Bisenzio, Italy).

### Trans-differentiation: adipogenic, osteogenic, and pancreatic

For adipocyte differentiation, cells were plated in AdipoDiff medium (Miltenyi Biotec GmbH, Bergisch Gladbach, Germany) for 3 weeks with medium change every 4 days. The accumulation of fat deposit was stained using Nile Red, a specific intercellular lipid staining, and the up-regulation of gene PPARG (peroxisome proliferator-activated receptor gamma) was quantified using RTqPCR. For osteoblast differentiation, cells were plated in OsteoDiff medium (Miltenyi Biotec) for 2 weeks. Alkaline phosphatase activity was detected using Sigma Fast BCIP/NBT substrate (Sigma-Aldrich) according to the manufacture’s protocol. The up-regulations of bone-specific genes were quantified using RTqPCR. The protocol for pancreatic cells differentiation was performed according previous reports in the presence of 10 mM nicotinamide [12,13]. The identification of cells was performed using RTqPCR on gene somatostatin (SST) and gastric inhibitory polypeptide (GIP). Direct DNA sequencing was carried out to confirm the result (automated sequencer ABI Prism 3500xl genetic analyser, Applied Biosystems, Monza, Italy).

### Co-culture with HCC cells

A total 50,000 cells/mL of CAF were co-cultured together with 100,000 cells/ml of HuH-7 and JHH-6 cells for 7 days in a 6-well plate of Transwell system (Corning Costar, Milan, Italy). The alteration of tumor promoting factors was evaluated by way of RTqPCR. For comparison, co-culture between NTF and both HCC cell lines was also performed in the similar condition. The experiment was conducted in duplicates in two independent sets.

### Xenograft assay

Male 7 weeks athymic nude (Foxn1(nu/nu)) homozygotes and NOD/SCID (NOD.CB17-*Prkdcscid*/NCrHsd) mice for *in vivo* xenotransplantation studies were obtained from Harlan Laboratories, Srl (Udine, Italy). All animals were maintained in the animal facility of the University of Trieste. After detachment, CAF was suspended in 100 μL cold PBS and placed in ice. A total of 50,000 to 1 million cells were injected subcutaneously into abdomen of the nude mouse in duplicates or orthotopic in the liver of NOD/SCID mice. Viability of the cells was checked by tryphan blue staining dye after injection. The xenotransplantated mice together with control were observed for four months after injection. Mouse body weight was measured every week. Serum alanin transferase (ALT) and aspartate transferase (AST) levels on sacrifice day were measured by photometric enzymatic test (Cobas, Roche, Mannheim, Germany).

### Statistical analysis

Students’ *t* test was performed for statistical comparison between groups using software InStat Version 3.05 (GraphPad Software, Inc., La Jolla, CA, USA). Statistical significance was set to p < 0.05.

## Results

### Morphology and characterization of the cells

**Figure 1:**
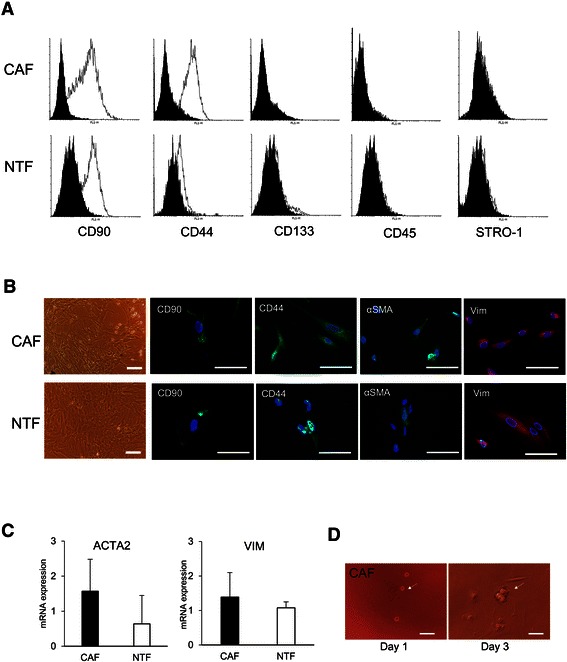
**The phenotypes of primary cells CAF and NTF. A**. Flow cytometric analysis of surface marker proteins showed the positivity for CD90 and CD44, and the negativity for CD133, CD45, and STRO-1. Black graph: control, White graph: positive expression. **B**. Morphology of the cells and localization of positive proteins CD90, CD44, αSMA, and Vimentin. Scale bar, 100 μm. **C**. mRNA expressions of vimentin (VIM) and αSMA (ACTA2) in CAF and NTF. VIM and ACTA2 mRNA relative expressions were normalized to reference genes RNA18S and ACTB. **D**. 3D clonogenic capacity of the CAF in Matrigel. Scale bar, 100 μm.

Primary cells from HCC (CAF) and from cirrhotic tissue (NTF) were obtained. All cells showed fibroblastic-like morphology and they had the ability to form clonal colonies after plating in low density. The presence of surface markers proteins was detected by flow cytometry and immunofluorescence. CAF showed higher percentages of antigens CD90 and CD44 (52 ± 27% and 59 ± 22%, respectively) as compared to NTF (37 ± 28% and 74 ± 12%), however the differences were not statistically significant. Hematopoietic cells markers CD133, CD45, and STRO-1 were either negative or very low expressed (Figure 1A).

The localizations of positive proteins CD90, CD44, Vim, and αSMA were visualized by immunofluorescence. As expected, CD90 and CD44 proteins were mainly localized on the cell membrane while αSMA and Vim in the cytoplasm (Figure 1B). When we compared the mRNA expression of the αSMA (ACTA2), we observed that ACTA2 expression was higher in CAF compared to NTF, but less noticeable for VIM (Figure 1C).

To assess the clonogenic capacity of the CAF as one of the characteristics of stem cells, CAF was grown in a 3-dimensional anchorage-independent matrix in Matrigel. As shown on Figure 1D, a single cell formed a colony three days after plating.

The presence of other cell markers was identified using RT-PCR, based on the consensus for the mesenchymal stem cells (MSC) and previous reports of multipotent hepatic stem cells [12,14]. As expected, qualitative PCR result showed that both CAF and NTF were positive for MSC markers CD90, CD44, CD29, CD13, CD105 and CD166, and negative for hematopoietic cells markers CD34, CD117, and CD45 (Table 1).

**Table 1:** **The genotypes of the primary cells**

	CAF		NTF	
	+	-	+	-
MSC requirement	CD90*	CD11B*	CD90*	CD11B*
	CD44	CD14*	CD44	CD14*
	CD29*	CD19*	CD29*	CD19*
	CD13	CD79*	CD13	CD79*
	CD105*	CD117	CD105*	CD117
	CD166	CD34*	CD166	CD34*
		CD45*		CD45*
Mesenchymal marker	ACTA2		ACTA2	
	VIM		VIM	
Pluripotency markers	OCT4		OCT4	
	SOX2		SOX2	
Reference	18S		18S	
	ACTB		ACTB	

The mRNA analysis by RT-PCR of consensus markers of MSC, mesenchymal activation, and pluripotency factors. + = positive expression, − = negative expression. *The reference standard based on the Mesenchymal and Tissue Stem Cell Committee of the International Society for Cellular Therapy [14].

### Adipogenic, osteogenic, and pancreatic trans-differentiation

**Figure 2:**
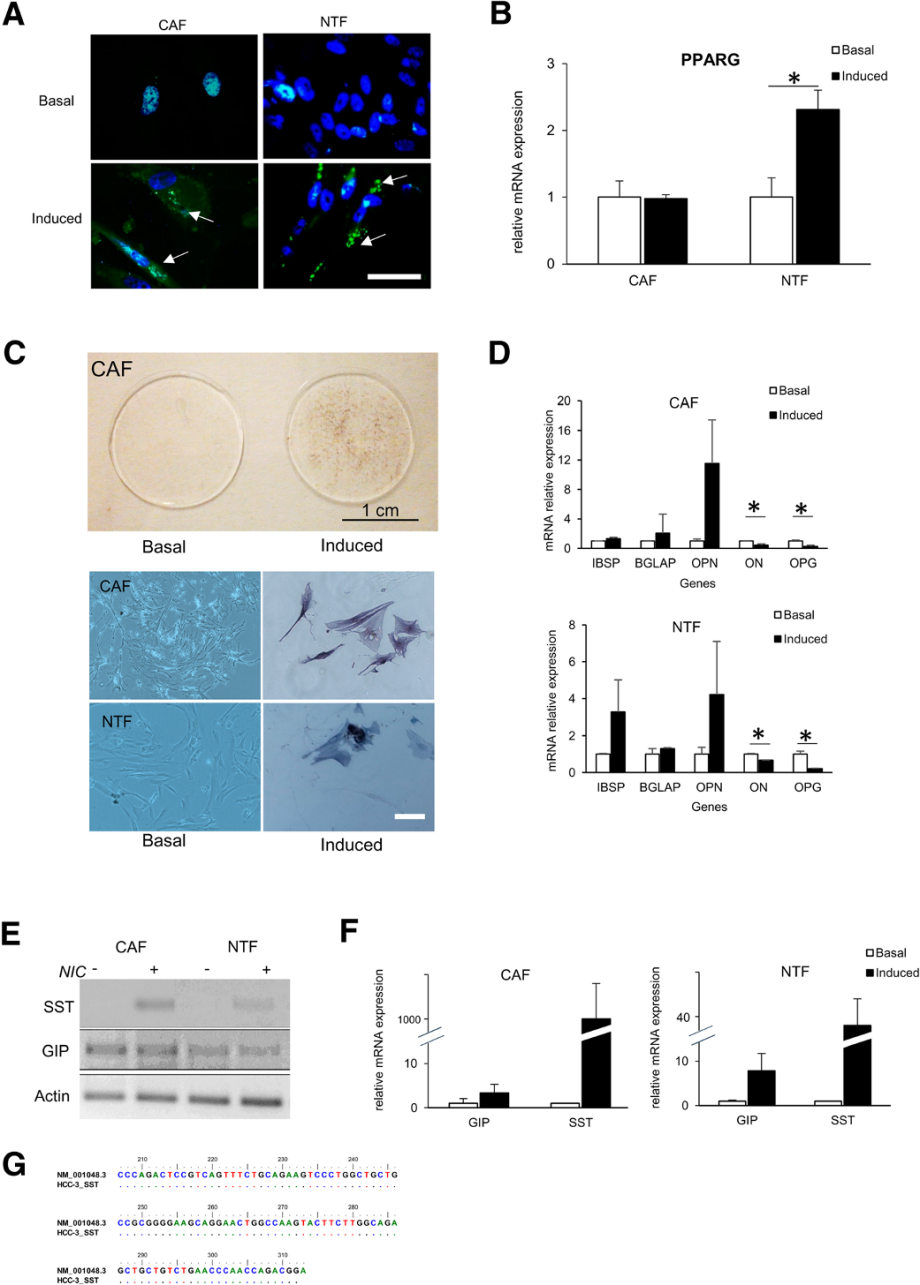
**Cells-directed*in vitro*differentiation of the CAF and the NTF. A-B**. Adipogenic differentiation. Nile Red staining of lipid deposition (arrow indicated) in the cytoplasm *(A)*. RTqPCR result showed the mean of PPARG gene regulation *(B)*. **C-D**. Osteogenic differentiation. Alkaline phosphatase staining of macro- (upper panel) and microscopic visualization (lower panel). Scale bar, 100 μm *(C)*. RTqPCR result of osteogenic differentiation genes osteocalcin (BGLAP), bone sialoprotein (IBSP), osteopontin (OPN), osteoprotegerin (OPG) and osteonectin (ON) *(D)*. **E-G**. Endodermic differentiation. The qualitative result of the induction of somatostation (SST) and gastric inhibitory protein (GIP) using gel electrophoresis *(E)*. RTqPCR result of endodermic differentiation genes showed an induction/up-regulation of SST and GIP. NIC = nicotinamide. The target mRNA expression was normalized to reference gene β-actin *(F)*. Confirmation by DNA sequencing of human SST. SST_HCC3 = DNA sequences of SST from induced CAF, NM_001048.3 = reference DNA sequences from GenBank *(G)*. For RTqPCR data, basal expression was considered as 1.00, target mRNA expressions were normalized to reference genes RNA18S and ACTB. Scale bar, 100 μm. *p < 0.05.

In total, nine primary cells CAF and NTF were subjected to adipogenic, osteogenic, and pancreatic *in vitro* differentiation in inducer medium. In adipogenic differentiation, by using Nile Red staining we observed lipid depositions in cytoplasm in five out of six samples induced (83%), in both CAF and NTF. However, a significant PPARG mRNA up-regulations was noticed only in NTF differentiation, with the mean increase of 2.31 ± 0.29 fold compared to basal level (p < 0.05). Induced CAF did not show an increment in PPARG mRNA expression (Figure 2A-B).

In osteogenic differentiation, induced CAF and NTF were positive for alkaline phosphatase staining. CAF and NTF showed similar tendencies on the regulation of bone-specific genes, with clear up-regulations of bone sialoprotein (IBSP), osteocalcin (BGLAP) and osteopontin (OPN) and down-regulations of osteonectin (ON) and osteoprotegerin (OPG) (p < 0.05) (Figure 2C-D).

When CAF and NTF were subjected to trans-differentiation into pancreatic cells with the presence of 10 mM nicotinamide, induced CAF and NTF showed a clear up-regulation of GIP with the means of mRNA increase of 3.3 ± 2.0 fold and 7.9 ± 10.1 fold, respectively. Moreover, SST mRNA was found to be strongly increased after the induction. DNA sequencing confirmed the specificity of SST DNA sequence (Figure 2E-G).

### Co-culture study

To investigate the difference between CAF and NTF in hepatocarcinogenesis and to explore the cross talk between these cells with HCC cells, a co-culture study in a Transwell system was performed. The HuH-7 and JHH-6 HCC cell lines were chosen to represent well- and poor-differentiated HCC cells, respectively. mRNA analysis showed that in basal condition, both CAF and NTF expressed higher level of fibroblast activated protein (FAP), ACTA2, and collagen type 1 (COL1) compared to those of HuH-7 and JHH-6 (p < 0.01 for all genes; data not shown).

**Figure 3:**
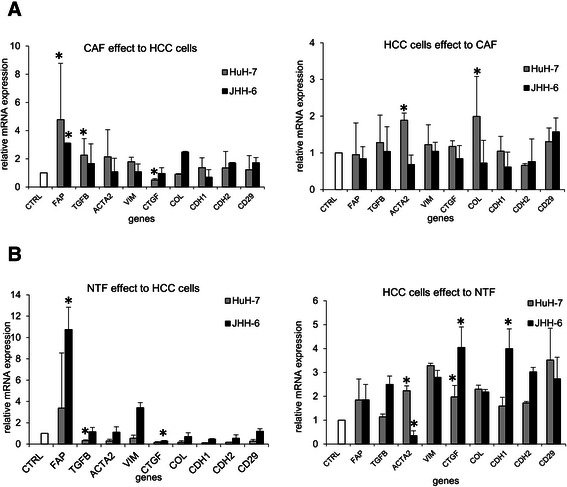
**Co-culture study between the primary cells and the HCC cell lines HuH-7 and JHH-6. A**. Cross-talk between the HCC cells lines and the CAF. **B**. Cross-talk between the HCC cells lines and the NTF. Target mRNA expression was normalized to reference genes RNA18S and ACTB. CTRL = cells control without co-culture (=1.00). Target mRNA expressions were normalized to reference genes 18SRNA and ACTB. *p < 0.05 compared to each control.

After co-culture, we observed a significant effect of the presence of both CAF and NTF in HCC cell lines. In the presence of the CAF, the up-regulations of TGFB1 and FAP were observed in both HCC cell lines (p < 0.05), a slight up-regulation of ACTA2 was observed in HuH-7. On the other hand, the presence of HuH-7 cells increased the expressions of ACTA2 and COL1 in CAF, while JHH-6 cells did not (Figure 3A).

In contrast, we noticed clear decrease of tumor supporting genes in HCC cells upon co-culture with the NTF with the exception of the increases of FAP and VIM in JHH-6. The mRNA expressions of CTGF, COL1, E-cadherin (CDH1), and N-cadherin (CDH2) were down-regulated in both HCC cell lines, while TGFB1 (p < 0.05), ACTA2, and integrin β1 (CD29) were down-regulated in HuH-7. In parallel, the presence of HCC cells up-regulated the expressions of CTGF (p < 0.05), VIM, COL1, and CD29 of the NTF. The expressions of TGFB1, CDH1, and CDH2 of the NTF were up-regulated after co-culture with JHH-6, a poor-differentiated HCC cell line (Figure 3B).

### Xenograft assay

**Figure 4:**
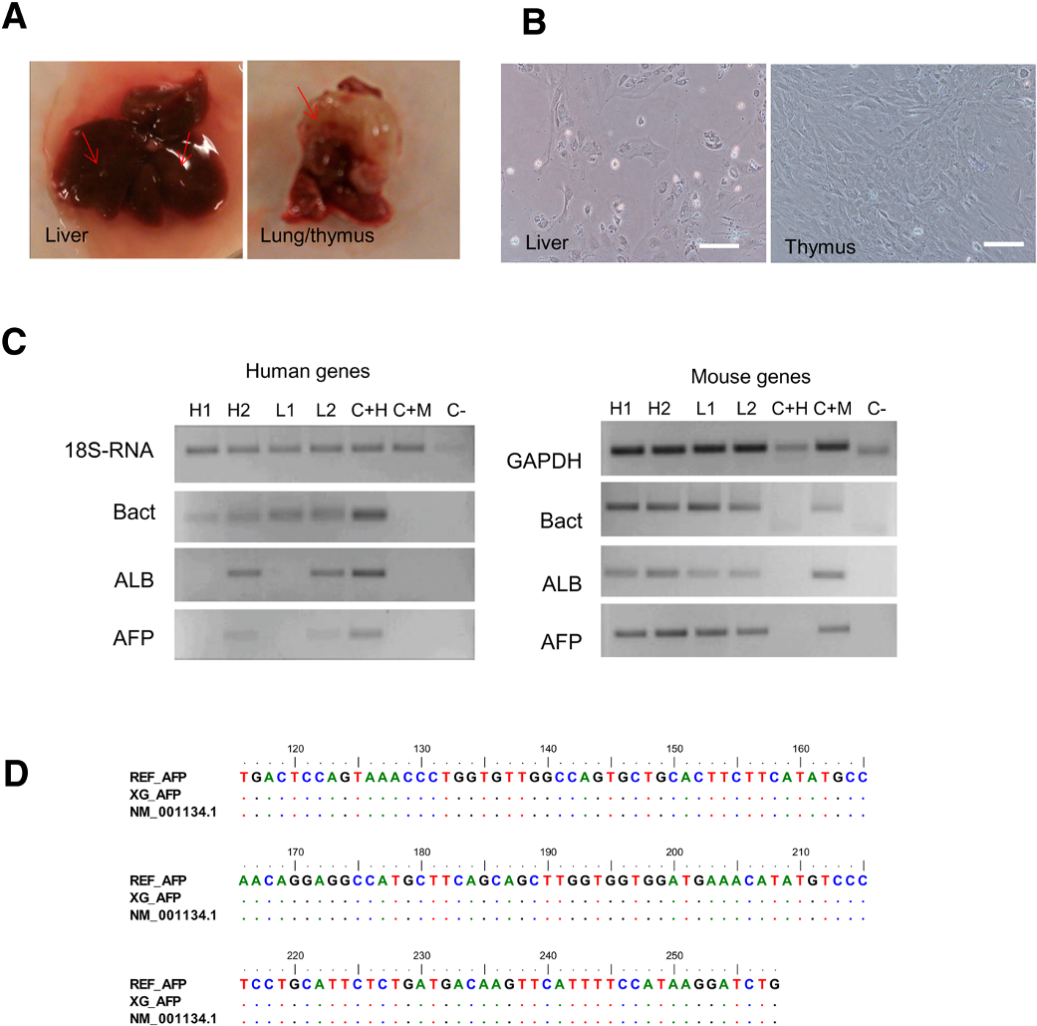
**Xenograft assay of the CAF. A**. Tissue mass in the liver and lung of the NOD/SCID mouse following orthotopic injection of the CAF from HCC. Arrow indicates nodules. **B**. Primary cells of the nodules in the orthotopic xenografts. Scale bar, 100 μm. **C**. The positivity of genes in the xenograft primary cells. H = hepatic, L = lung, C + H = positive control human (human liver), C + M = positive control mouse (mouse liver). Gene 18S is homolog for human and mouse species. **D**. DNA sequencing of human AFP of primary cells obtained from the lung of xenograft. REF_AFP = DNA sequences of AFP genes from injected CAF, XG_AFP = DNA sequences of AFP genes from primary cultures of xenograft, NM_001134.1 = reference DNA sequences from GenBank.

Two CAFs were subjected to xenograft assay. The viability of the cells was more than 95%. Four months after subcutaneous injection in nude mice, the serum level of ALT and AST of xenografts were slightly increased compared to control (102 ± 23 IU vs. 77 ± 9 IU for ALT and 23 ± 2 IU vs. 17 ± 1 IU for AST) even though no tumor nodules were observed. Orthotopic injection in NOD/SCID mice resulted in the appearance of nodules in the liver as well as in the lung and thymus. Cultured primary cells of the nodules and the injected sites expressed liver-specific markers ALB and AFP, including those of human, as confirmed by DNA sequencing with reference to DNA sequences of the CAF (Figure 4). However, we could not notice the human CD90 protein. The positivity of human and murine genes of xenograft models is listed on Table 2. No difference in body weight of all animals was noticed (data not shown).

**Table 2:** **The positive expression of human and mouse genes of the xenograft tissues**

Strain	Injection site	n	Nodule	Human genes +	Mouse genes +
Nude mice	Subcutaneous	3	N/D	ALB, AFP, Actin, 18S	Afp, Actin, Gapdh, 18S
NOD/SCID mice	Intrahepatic	2	liver and lung	ALB, AFP, Actin, 18S	Afp, Alb, Actin, Gapdh, 18S

Human and mouse genes positivity were detected using RT-PCR on xenograft tissues. Species specificity was checked using BLAST of NCBI. N/D: not detectable.

## Discussion

We report on the presence of CAF and NTF populations with the characteristic of MSC in human hepatic tissues obtained from HCC patients. The CAF and NTF were negative or low expressed for the markers of endothelial (CD31) and hematopoietic cells (CD34, CD45). They had spindle-shape fibroblast-like morphology with the capacity to form colonies on plastic surface, and for CAF in three-dimensional matrix, indicating its anchorage-independent capacity and clonogenicity. The morphologic and phenotypic characteristics of the cells were maintained during subcultures.

Previously, Herrera *et al.* reported the isolation of the multipotent hepatic liver stem cells (HLSC) from normal human liver expressing MSC markers, vimentin, and nestin. They could be differentiated not only into mesodermal lineages, but also into endodermal lineage [12]. In this work, we induced both CAF and NTF into mesodermal lineage (adipocytes and osteoblasts) and endodermal lineage (pancreatic cells). After adipogenic induction, lipid deposition in cytoplasm was clearly noticeable, accompanied by the up-regulation of PPARG, a master regulator of adipocyte differentiation, in the NTF. After osteogenic induction, the cells were positive for alkaline phosphatase staining as well as the up-regulations of bone-specific genes. Our data is in line with a previous study by Cesselli *et al*. that the multipotent adult stem cells from diseased liver cultured in osteogenic medium became positive to von Kossa staining specific for bone differentiation and expressed osteocalcin [6].

To investigate whether the cells can be differentiated into not only similar mesenchymal lineage but also more advanced into endodermal lineage, we induced the cells into pancreatic cells. After induction, we observed the induction of SST, markers for δ cell, and the up-regulation of GIP, a member of glucagon, marker of α cell. However, the expression of insulin, a marker for β cells of pancreatic islet, was either absent of too low to be detected, at least at mRNA level. One possible explanation is that these cells were derived from cirrhotic and HCC tissues and their multipotent capacity might be more restricted compared to that of normal HLSC. Both phenotype and capacity of these cells are in agreement with the consensus of the Mesenchymal and Tissue Stem Cell Committee of the International Society for Cellular Therapy [14,15]. In particular, they showed plastic adherence in standard culture condition, expression of CD105 and CD90, but not CD45 and CD34, and the potency to differentiate into mesenchymal lineage *in vitro.*

From the trans-differentiation data, we found that both CAF and NTF had comparable potential to be induced into various cell types, even though the plasticity of the NTF was higher than the CAF. To understand their intrinsic difference in hepatocarcinogenesis, we performed a co-culture experiment with HCC cell lines with different degrees of differentiation, in a Transwell system. We observed that the presence of the CAF increased the expression of several genes involved in tumorigenesis in HCC cells. The expression of TGFB1 was increased, particularly in HuH-7, and a very strong up-regulation was observed for the FAP in both HCC cell lines.

In contrast, the up-regulation effect of HCC cells was apparent in the NTF, while the presence of the NTF down-regulated the factors of HCC. The presence of HCC cells induced a significant up-regulation of CTGF, as well as VIM, COL1, CDH2, and CD29, markers for mesenchymal phenotypes. This result was in accordance with previous data observed by Mazzocca *et al*. that HCC invasive cells produced high level of CTGF and they generated tumors with high stromal component *in vivo* [4]. The up-regulation ACTA2 of the NTF in the presence of HuH-7 is also in agreement with the previous report on the trans-differentiation of the PTF into CAF-myofibroblast in the presence of conditioned medium of HuH-7, even though in our experimental set we did not add the LPA [5]. In this study, the extent of the mRNA expressions in the NTF was higher in the presence of the poor-differentiated HCC cells JHH-6 compared to well-differentiated HuH-7 cells. These data support our conclusion on the mutual cross talk of the CAF, the NTF, and the cancer cells.

Yang *et al.* had demonstrated that CD90 cells isolated from primary HCC induced tumor and lung metastasis after orthotopic injection to SCID/Beige mice, indicating the CD90+ cells as a marker of cancer stem cells (CSC) [16]. Our *in vivo* data showed that subcutaneous injection of the CAF expressed CD90+ failed to induce tumor in nude mice four months after injection. However, orthotopic injection in the liver of NOD/SCID mouse induced mass in the liver and the lung. Interestingly, we noticed the presences of human ALB and AFP in the primary cells obtained from the lung mass. We assumed that the CAF were able to enter into circulation and interact with resident tissue environment, this indirectly supported our co-culture data *in vitro*. However, we did not found cells positive for human CD90 and CD44 in both liver and lung tissue. Therefore, although this initial *in vivo* data is important, further studies must be conducted to prove this interaction.

The negativity of human HCC after injection might indicate either different function of CD90 cells population in HCC or the presence of several different populations of CD90 phenotype. We had recently reported that the CD90 was up-regulated in liver cirrhosis and HCC compared to normal tissue [17] and data from Yamashita *et al.* had shown that the tumorigenicity of the CSC CD90+ cells might appear in the late stages of hepatocarcinogenesis with the preference to HBV-related HCC [18]. In our study, none of our CAF was derived from HBV-related HCC and the tissues were obtained from early stage of the disease undergoing partial hepatectomy. It is possible that it might be one of the reasons of this discrepancy, even though we are not in the position to demonstrate this point further.

Although the origin of CAF in HCC is still controversial, our result showed that CAF was able to differentiate into other cell types and to circulate to other organs, one of the characteristics of mesenchymal or stromal stem cells. On the other hand, CAF could be also derived upon a paracrine mechanism between multipotent NTF and HCC cells. Further studies will be essential to demonstrate the plasticity and mobility of resident fibroblasts in normal liver as well as in the progression of liver disease.

## Conclusion

In conclusion, our data provides clear evidence of the plasticity of the stem cells-like CAF and NTF isolated from HCC and liver cirrhosis in the process of hepatocarcinogenesis and metastasis. These cells mutually interact with HCC cells and deregulate important tumor promoting factors. Their trans-differentiation flexibility may induce a switch from normal to cancerous microenvironment.

## Additional file

Additional file 1: Table S1. List of primers.

## Abbreviations

ACTA2: Alpha smooth muscle actin
ALT: Alanin transferase
AST: Aspartate transferase
BGLAP: Osteocalcin, bone gamma-carboxyglutamate protein
CAF: Cancer associated fibroblast
CD: Cluster of differentiation
COL1: Collagen type 1
CSC: Cancer stem cells
CTGF: Connective tissue growth factor
CTRL: Control
DNA: Deoxyribonucleic acid
GIP: Gastric inhibitory polypeptide
HBV: Hepatitis B virus
HCC: Hepatocellular carcinoma
HCV: Hepatitis C virus
IBSP: Bone sialoprotein
mRNA: Messenger ribonucleic acid
MSC: Mesenchymal stem cells
NTF: Non-tumoral fibroblast
ON: Osteonectin
OP: Osteopontin
OPG: Osteoprotegerin
PLC: Primary liver cancer
PPARG: Peroxisome proliferator-activated receptor gamma
RNA: Ribonucleic acid
RTqPCR: Reverse transcription quantitative polymerase chain reaction
SC: Stem cells
SST: Somatostatin
TGFB: Transforming growth factor beta
