# Supplementary material for: The role of multipotent cancer associated fibroblasts in hepatocarcinogenesis
Source: BMC Cancer. 2015 Mar 27;15:188. doi: 10.1186/s12885-015-1196-y (PMC4389787; doi:10.1186/s12885-015-1196-y)
Supplement: Additional file 1: Table S1. — List of primers. [file 12885_2015_1196_MOESM1_ESM.pdf]

Table S1. List of primers

Gene	Acc. No.	Sequence F	Sequence R	Ref.
Reference genes				
18S	NR_003286.2	TAACCCGTTGAACCCCATT	CCATCCAATCGGTAGTAGCG	[1]
ACTB	NM_001101.3	CGCCGCCAGCTCACCATG	CACGATGGAGGGGAAGACGG	TS
Cells characterization				
AFP	NM_001134	GCGGCTGACATTATTATCG	TTGGCACAGATCCTTATGG	TS
ALB	NM_000477.3	GGCATCCTGATTACTCTGTCG	AATTCTGAGGCTCTTCCACAAG	TS
CD105	NM_001114753.1	CTTCCTCCTCCACTTCTAC	GGACTTCCTGGTCTTGAG	TS
CD117	NM_000222	AGGCTCTTCTCAACCATCTG	ATTCATTCTGCTTATTCTCATTCG	TS
CD11B	NM_000632.3	AGAATCCAGTGTGACATC	GTTATGCGAGGTCTTGAT	TS
CD13	NM_001150	TACCATCATCAGCATTAC	CACCACCATAATCGTTAA	TS
CD133	NM_006017	CATCTGCTCTCTGCTGAC	AACTTAATCCAACTCCAACC	TS
CD14	NM_001040021.2	TTCACAATCTCTTCCACAA	CATTTCTTTGACTTCTTCCTT	TS
CD166	NM_001627.2	GAAGGAGGAGGAATATGGAATC	GTCAAGTCGGCAAGGTATG	TS
CD19	NM_00178098.1	GATTCACACCTGACTCTG	ACACATCCTAAGCAACATT	TS
CD29	NM_002211.3	GCCTTGGTGTCTGTGCTGAG	AGTCGTCAACATCCTTCTCCTTAC	TS
CD31	NM_000442	GAGTCCAGCCGCATATCC	TGACACAATCGTATCTTCCTTC	TS
CD34	NM_001025109	CTGATACCGAATTGTGACTC	TTGGGCGTAAGAGATGTC	TS
CD44	NM_000610	CTCATACCAGCCATCCAATG	GAGTCCATATCCATCCTTCTTC	TS
CD45	NM_002838.3	CGGCTGACTTCCAGATATGAC	GCAGTGGTGTGAGTAGGTAAG	TS
CD79	NM_001783.3	ATTGTAGCAGCCTCGTTA	CAGTTGGGAAGAATTTATTATCAC	TS
CD90	NM_006288.2	AGAGACTTGGATGAGGAG	CTGAGAATGCTGGAGATG	TS
CK19	NM_002276.4	TGAGTGACATGCGAAGCCAATAT	GCGACCTCCCGGTTCAAT	[2]
CK7	NM_005556.3	TGAATGATGAGATCAACTTCCTCAG	TGTCGGAGATCTGGGACTGC	[2]
EpCAM	NM_002354.1	GAATAATAATCGTCAATGCCAGTG	CGCTCTCATCGCAGTCAG	TS
SOX2	NM_003106.2	AGCTACAGCATGATGCAGGA	GGTCATGGAGTTGTACTGCA	[3]
OCT4	NM_002701	AGCGAACCAGTATCGAGAAC	TTACAGAACCACACTCGGAC	[3]
Cells differentiation				
PPARG	NM_138712.3	ACGAAGACATTCCATTCACAAG	TCTCCACAGACACGACATTC	TS
GIP	NM_004123.2	CCCTCAACCTCGAGGCCCCA	CCCGAGCCTCCCTCTGGGTG	TS
SST	NM_001048.3	CCCAGACTCCGTCAGTTTCT	AGCCTGGGACAGATCTTCAG	TS

BGLAP	NM_199173.4	CAGAGTCCAGCAAAGGTG	AGCCATTGATACAGGTAGC	TS
IBSP	NM_004967.3	CGAGCCTATGAAGATGAG	GTGGTGGTAGTAATTCTGA	TS
OP	NM_001040058	AATGATGAGAGCAATGAG	GTCTACAACCAGCATATC	TS
OPG	NM_002546	AATGTGGAATAGATGTTACC	TCTACCAAGACACTAAGC	TS
ON	NM_003118	GTATCTGTGGGAGCTAATCCT	AGAGTCGAAGGTCTTGTTGTC	[4]
Tumor promoting factors				
FAP	NM_004460.2	TATTCAGAGTAACACAGGATTCA	ACTTCTTGCTTGGAGGATAG	TS
TGFβ1	NM_000660.4	GCAACAATTCCTGGCGATACC	CTCCACGGCTCAACCACTG	TS
CTGF	NM_001901.2	AGACCTGTGCCTGCCATTAC	TGTGAATCAGTTCAAGTTCCA GTC	TS
ACTA2	NM_001141945	TTGGCTTGGCTTGTCAGG	GCTTTAGGGTCGCTGGAG	TS
VIM	NM_003380.3	AACTTCTCAGCATCACGATGAC	TTGTAGGAGTGTCGGTTGTTA AG	TS
CDH1	NM_004360.3	GGAACTATGAAAAGTGGGCTTG	AAATTGCCAGGCTCAATGAC	[5]
CDH2	NM_001792.3	GACGGTTCGCCATCCAGAC	TCGATTGGTTTGACCACGG	[6]
COL1	NM_000088	GGCTCCTGCTCCTCTTAG	GTGGGATGTCTTCGTCTTG	TS

References

1. Schmittgen TD, Zakrajsek BA: **Effect of experimental treatment on housekeeping gene expression: validation by real-time, quantitative RT-PCR**. *J Biochem Biophys Methods* 2000, **46**:69–81.

2. Dimmler A, Gerhards R, Betz C, Günther K, Reingruber B, Horbach T, Baumann I, Kirchner T, Hohenberger W, Papadopoulos T: **Transcription of cytokeratins 8, 18, and 19 in bone marrow and limited expression of cytokeratins 7 and 20 by carcinoma cells: inherent limitations for RT-PCR in the detection of isolated tumor cells**. *Lab Investig J Tech Methods Pathol* 2001, **81**:1351–1361.

3. Park I-H, Zhao R, West JA, Yabuuchi A, Huo H, Ince TA, Lerou PH, Lensch MW, Daley GQ: **Reprogramming of human somatic cells to pluripotency with defined factors**. *Nature* 2008, **451**:141–146.

4. Dass CR, Ek ET, Contreras KG, Choong PF: **A novel orthotopic murine model provides insights into cellular and molecular characteristics contributing to human osteosarcoma**. *Clin Exp Metastasis* 2006, **23**:367–380.

5. Lin C-W, Liao M-Y, Lin W-W, Wang Y-P, Lu T-Y, Wu H-C: **Epithelial cell adhesion molecule regulates tumor initiation and tumorigenesis via activating reprogramming factors and epithelial-mesenchymal transition gene expression in colon cancer**. *J Biol Chem* 2012, **287**:39449–39459.

6. Kroepil F, Fluegen G, Totikov Z, Baldus SE, Vay C, Schauer M, Topp SA, Esch JSA, Knoefel WT, Stoecklein NH: **Down-regulation of CDH1 is associated with expression of SNAI1 in colorectal adenomas**. *PloS One* 2012, **7**:e46665.
